# Patient-Reported Perceptions of Nasal Aesthetic and Functional Changes Following Le Fort I Osteotomy: A Postoperative Survey-Based Study

**DOI:** 10.3390/healthcare14060812

**Published:** 2026-03-22

**Authors:** Nuri Can Tanrısever, Mehmet Okan Akçam

**Affiliations:** Department of Orthodontics, School of Dentistry, University of Ankara, Ankara 06560, Türkiye

**Keywords:** Le Fort 1 osteotomy, nasal aesthetic, breathing function, survey

## Abstract

**Highlights:**

**What are the main findings?**

- Le Fort I osteotomy is associated with improved patient-reported perceptions of nasal aesthetics and breathing.
- Postoperative perception scores were consistently higher than retrospective preoperative scores across all survey items.

**What are the implications of the main findings?**

- Patient-reported outcome measures provide valuable insight beyond objective assessments in orthognathic surgery.
- Preoperative counseling should address potential nasal aesthetic and breathing changes to align surgical outcomes with patient expectations.

**Abstract:**

**Background/Objectives**: Le Fort I osteotomy, a commonly performed maxillary surgical procedure, is known to influence nasal aesthetics and perceived breathing function due to its close anatomical relationship with the nasal structures. While objective nasal changes have been extensively documented, patient-reported perceptions of aesthetic appearance and breathing remain clinically important for evaluating surgical success. This study aimed to assess patient-reported perceptions of nasal aesthetic and functional outcomes following Le Fort I osteotomy using a postoperative survey-based approach. **Materials and Methods**: This study included 200 patients (mean age: 25 ± 4.19 years) who underwent Le Fort I osteotomy at the Department of Orthodontics, Ankara University. Patient perceptions of nasal aesthetics and breathing were evaluated using a structured Likert-scale questionnaire administered six months postoperatively. Perceived preoperative conditions and current postoperative perceptions were assessed within the same survey. Statistical analysis was performed using SPSS version 26, with the significance level set at *p* < 0.05. **Results**: The findings demonstrated a statistically significant improvement in patient-reported satisfaction with nasal appearance and perceived breathing comfort following surgery. A greater proportion of patients reported increased satisfaction with their nasal aesthetics, believed that others viewed their nasal appearance more positively, and experienced improved nasal breathing in the postoperative period. **Conclusions**: Le Fort I osteotomy was associated with positive patient-reported perceptions of both nasal aesthetics and breathing function. However, individual anatomical characteristics and patient expectations appear to influence the perceived outcomes. These findings underscore the value of incorporating patient-reported outcome measures into preoperative counseling and postoperative evaluation in orthognathic surgery.

## 1. Introduction

Orthognathic surgery is a well-established treatment modality that involves the surgical repositioning of the jawbones to correct dentofacial deformities. This approach aims to eliminate skeletal and dental discrepancies, enhance facial harmony, and restore functional balance, thereby contributing to both aesthetic improvement and functional rehabilitation [1].

Movements of the jawbones in the sagittal, vertical, or transverse directions inevitably result in changes in the overlying soft tissues, including the nose, lips, chin, and cheeks. Previous studies have reported that soft tissue responses may account for approximately 60–90% of the corresponding skeletal movements, although this proportion varies depending on the anatomical region involved [2]. Because postoperative soft tissue contours play a decisive role in perceived aesthetic outcomes, understanding the relationship between skeletal repositioning and soft tissue response is essential for successful orthognathic treatment planning and evaluation [3].

Among the facial structures, the nose occupies a central position and plays a critical role in facial aesthetics. Due to its close anatomical relationship with the maxilla, the nasal region is directly affected by Le Fort I osteotomy, which is commonly performed in maxillary surgery. Changes in nasal base width, nasal tip position, and the nasolabial angle following Le Fort I osteotomy have been well-documented in the literature [4,5,6]. In contrast, mandibular surgery has been reported to have a limited direct effect on nasal morphology; however, it may indirectly influence nasal projection by altering the nose–chin relationship [7,8,9]. These nasal aesthetic changes can substantially affect patients’ self-evaluation and satisfaction, highlighting the importance of careful preoperative assessment and patient counseling [10].

In addition to aesthetic improvements, orthognathic surgery has been shown to significantly enhance quality of life by improving chewing, speech, and breathing functions. The literature reports favorable outcomes following orthognathic procedures with respect to masticatory efficiency, speech articulation, and breathing function [11]. Specifically, maxillary advancement and/or impaction achieved through Le Fort I osteotomy has been associated with expansion of the nasal floor and upper airway, resulting in reduced nasal resistance and improved breathing comfort [12,13,14].

While objective assessments of skeletal and soft tissue changes after orthognathic surgery are well-represented in the literature, the evaluation of patients’ own perceptions of these changes remains relatively limited. Patient-reported outcome measures provide valuable insight into how individuals experience surgical results and are increasingly recognized as an integral component of treatment success. However, despite the abundance of studies objectively assessing nasal changes, relatively few investigations have focused on patients’ subjective perceptions of nasal aesthetics and breathing function.

Accordingly, the aim of this study was to evaluate patient-reported perceptions of nasal aesthetic appearance and breathing function associated with Le Fort I osteotomy using a survey-based approach.

## 2. Materials and Methods

This study included 200 patients (mean age: 25 ± 4.19 years; 85 males and 115 females) who had undergone orthodontic treatment combined with Le Fort I osteotomy at the Department of Orthodontics, Faculty of Dentistry, Ankara University. Participation in the survey component of the study was voluntary.

All orthognathic procedures were performed by the same maxillofacial surgery team following a standardized protocol. In all patients, Le Fort I osteotomy was carried out for maxillary repositioning according to individual skeletal diagnosis. The surgical movements included maxillary advancement, maxillary impaction, or a combination of both, depending on the underlying sagittal and vertical skeletal discrepancies. Of the total sample, 135 patients (67.5%) underwent isolated Le Fort I osteotomy, whereas 65 patients (32.5%) underwent bimaxillary surgery including concomitant mandibular osteotomy (bilateral sagittal split osteotomy for mandibular advancement or setback). The distribution of surgical procedures has been added to Table 1.

Patient-reported perceptions of nasal aesthetics and breathing function were evaluated using a researcher-developed questionnaire administered six months postoperatively. At the time of survey administration, no persistent postoperative hypoesthesia was documented in the clinical records. Within the same survey, participants were asked to retrospectively assess their perceived preoperative condition as well as their current postoperative perceptions.

Ethical approval for the study was obtained from the Non-Clinical Scientific Studies Ethics Committee of Ankara University, Faculty of Dentistry (Meeting No: 14; Approval Date: 6 October 2025). Written informed consent was obtained from all participants prior to survey administration.

**Figure 1:**
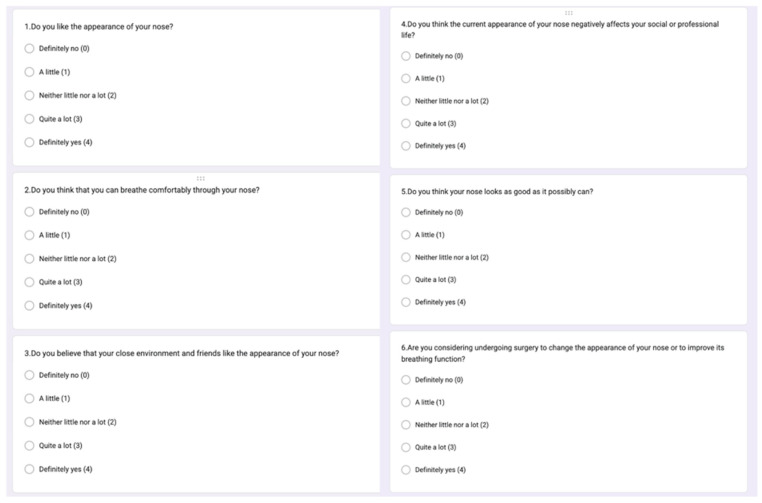
Survey questions.

The questionnaire consisted of an introductory section collecting demographic information, including age, sex, height, weight, and the presence of systemic diseases (Table 1). In the first section of the survey, participants responded to six questions assessing their perceived preoperative breathing function, nasal appearance, and the perceived impact of these factors on their social interactions. In the second section, participants answered the same six questions with reference to their current postoperative condition (Figure 1). Responses were recorded using a five-point Likert-type scale (0 = definitely not, 1 = little, 2 = neither a little nor a lot, 3 = quite a lot, and 4 = definitely yes). The questionnaire items were designed to reflect key domains of patient-reported aesthetic and functional perception relevant to orthognathic surgery. These items were generated by the research team based on clinically relevant domains commonly discussed in orthognathic surgery outcomes, including perceived nasal appearance, breathing comfort, and the potential social impact of nasal aesthetics.

### Statistical Analysis

Statistical analysis was performed using SPSS software (version 26.0; IBM Corp., Armonk, NY, USA).

An a priori sample size calculation was conducted using G*Power 3.1 software based on the primary within-subject comparison between retrospectively assessed preoperative and current postoperative perception scores. Assuming a two-tailed test, an alpha level of 0.05, a statistical power of 0.90, and a moderate effect size (Cohen’s dz = 0.50), the minimum required sample size was calculated as 43 participants.

A moderate effect size was selected to represent a clinically meaningful shift corresponding approximately to a one-category change on the five-point Likert scale at the group level. The final sample of 200 patients substantially exceeded this requirement, indicating adequate power to detect clinically relevant differences.

Frequency and percentage distributions were calculated for categorical variables, while continuous variables were summarized using descriptive statistics (minimum, maximum, mean, and standard deviation).

Because retrospective preoperative and postoperative responses were obtained from the same participants, the data represent paired (within-subject) measurements. As the data did not meet normality assumptions, the non-parametric Wilcoxon signed-rank test was used for comparisons. Statistical significance was set at *p* < 0.05.

To complement *p*-values and enhance clinical interpretability, effect sizes were calculated using the formula r = Z/√N, where N represents the number of paired observations with non-zero differences. Effect sizes were interpreted as small (0.10), moderate (0.30), and large (0.50). Because the effect size was derived from the non-parametric Wilcoxon signed-rank test using the r statistic (r = Z/√N), confidence intervals were not calculated, as this metric does not have a straightforward confidence interval estimation within the analytical framework applied in this study.

Detailed quantitative data regarding the magnitude of maxillary advancement or impaction were not analyzed as independent predictors, as the present study focused on the overall patient-reported outcomes rather than movement-specific effects.

Because the survey was administered at a single standardized postoperative time point within a cross-sectional framework, test–retest reliability was not evaluated. However, all participants completed the questionnaire under standardized conditions.

## 3. Results

Descriptive distributions of preoperative and postoperative responses are presented in Table 2 and Table 3. Overall, postoperative responses demonstrated a consistent shift toward more favorable perceptions across the aesthetic and functional domains.

With respect to nasal aesthetics, the proportion of participants reporting high satisfaction increased markedly after surgery. Similarly, the perception that the nose looked “as good as it possibly could” showed a substantial upward shift in postoperative responses.

Regarding nasal breathing, postoperative responses indicated improved perceived breathing comfort, with a clear reduction in low-comfort responses and an increase in high-comfort categories.

Perceived social perception also improved postoperatively, with more participants reporting that their close environment viewed their nasal appearance positively. Conversely, perceived negative social or professional impact decreased following surgery.

Detailed distributions of the response frequencies and percentages are presented in Table 2 and Table 3.

Comparison of the median perception scores demonstrated statistically significant differences between the current postoperative perceptions and retrospectively assessed the preoperative perceptions. For positively worded items, postoperative scores were significantly higher than retrospectively reported preoperative scores. In contrast, for the negatively worded item assessing social or professional impact, postoperative scores were significantly lower, indicating a reduction in perceived negative impact following surgery (Wilcoxon signed-rank test, *p* < 0.001). Effect size analysis (r = Z/√n) indicated moderate to large effects for most items (r = 0.47–0.66), whereas the negatively worded social impact item demonstrated a small-to-moderate effect size (r = 0.24) (Table 4).

## 4. Discussion

The purpose of this survey-based study was to evaluate patient-reported perceptions of nasal aesthetics and breathing following Le Fort I osteotomy by comparing retrospective preoperative perceptions with current postoperative perceptions assessed at six months. Overall, statistically significant differences were observed between retrospectively assessed preoperative and current postoperative perceptions. For positively worded items, postoperative scores were higher, whereas the negatively worded item demonstrated lower postoperative scores, indicating reduced perceived negative impact. In addition to statistical significance, moderate to large effect sizes were observed for most items, supporting the clinical relevance of these perceived changes.

Individuals may seek orthognathic surgery for multiple reasons, including improving facial aesthetics as well as functional concerns such as chewing, speech, and breathing, or addressing TMJ-related complaints. When motivations for orthognathic surgery have been examined, aesthetics has been reported to be a more prominent driver than function [15]. Therefore, even when objective assessments suggest favorable outcomes, dissatisfaction with postoperative aesthetics may negatively influence overall patient satisfaction and perceived treatment success. In particular, interventions affecting the nasolabial region should be planned carefully, and soft tissue implications should be considered in surgical planning and patient counseling [16].

A substantial body of literature has evaluated patient satisfaction after orthognathic surgery. While many studies report overall positive satisfaction outcomes [17,18,19,20], dissatisfaction rates of approximately 15% have also been reported [21]. However, the number of studies specifically focusing on patient-reported perceptions of nasal aesthetics and breathing following Le Fort I osteotomy remains limited.

Le Fort I osteotomy can influence nasal aesthetics primarily through three-dimensional repositioning of the maxilla. Alar base widening—particularly when combined with maxillary impaction and/or advancement—has been reported as one of the most frequent nasal soft tissue changes after maxillary osteotomies [22,23,24,25,26]. The perceived direction of this change may vary depending on the preoperative facial characteristics and postoperative expectations. For example, when the interalar distance is initially narrow, some degree of widening may be perceived favorably, whereas uncontrolled alar base widening and upper lip thinning may be perceived negatively and compromise aesthetic satisfaction [27].

In addition, mandibular procedures that do not directly alter the nasal region may still influence perceived nasal appearance by modifying chin projection and overall facial balance [28]. Following mandibular setback, the nose may appear relatively more prominent, whereas mandibular advancement may increase lower facial projection, potentially reducing the perceived prominence of the nose. Therefore, potential postoperative changes that could adversely affect the perceived aesthetics should be anticipated during planning, and patients should be informed in detail preoperatively; adjunctive intraoperative measures may be considered when indicated.

In the present study, postoperative responses suggested improved patient-reported perceptions of nasal aesthetics. In particular, increases in “definitely yes” responses to the questions “Do you like the appearance of your nose?”, “Do you think your nose looks as good as it possibly can?”, and “Do you believe your close environment and friends like the appearance of your nose?” indicate higher satisfaction with nasal appearance after surgery. These findings are consistent with previous reports [29]. However, variability across studies should also be acknowledged. Differences in surgical techniques, the magnitude and direction of maxillary movements, the presence of concomitant mandibular procedures, and patient-specific anatomical characteristics may influence postoperative nasal morphology and patient perception. In addition, variations in study design, outcome assessment methods, and follow-up duration may contribute to differences in reported findings across the literature.

Nevertheless, despite statistically significant overall improvements, some participants continued to report dissatisfaction or perceived negative social impact after surgery. This variability is clinically important and should not be overlooked. Statistical significance at the group level does not imply uniform benefit at the individual level. Residual dissatisfaction may reflect differences in preoperative expectations, psychological factors, social context, or heightened aesthetic awareness following treatment. In certain cases, even subtle asymmetries or minor deviations from anticipated outcomes may influence subjective evaluation, despite measurable functional or structural improvement. These findings highlight that patient-reported outcomes following orthognathic surgery are inherently heterogeneous and reinforce the importance of individualized preoperative counseling and realistic expectation management.

Le Fort I osteotomy may also affect functional outcomes, including breathing. Maxillary advancement and/or impaction can alter the geometry of the nasal cavity and nasal base, potentially reducing nasal resistance and improving nasal breathing [30,31]. In contrast, it has been suggested that upper airway narrowing may occur following mandibular setback; therefore, perceived functional improvements may be more pronounced in cases undergoing isolated Le Fort I osteotomy or bimaxillary advancement [31,32,33]. Objective airway changes are often evaluated using volumetric measurements; however, objectively detected changes may not always correspond to patients’ subjective experiences. For this reason, patient-reported perceptions of breathing should be assessed alongside objective outcomes. While patient-reported breathing is generally reported to improve after Le Fort I osteotomy, some patients may also report nasal congestion in the postoperative period [13,34]. In addition, craniofacial morphology itself may influence functional respiratory perception. Previous research has shown that variations in skeletal craniofacial patterns may be associated with functional adaptations related to breathing and airway dynamics [35]. Therefore, patient-reported breathing outcomes following orthognathic surgery should be interpreted not only in relation to surgical movements, but also within the broader context of craniofacial structural characteristics.

In our study, there was a notable increase in “definitely yes” responses to the question “Do you think that you can breathe comfortably through your nose?”, along with a decrease in “definitely not” responses and a significant increase in the median score for this item (preoperative = 2, postoperative = 3). These findings indicate higher postoperative breathing perception scores compared with retrospectively assessed preoperative scores.

Patient-reported perceptions of nasal aesthetics and breathing may be influenced by the direction and magnitude of skeletal movements, concomitant nasal procedures (e.g., septoplasty or turbinectomy), and postoperative edema. In the present study, the absence of additional nasal procedures and the timing of the survey at the sixth postoperative month may have facilitated a clearer perception of changes. Nevertheless, concomitant mandibular surgery in some participants remains a potential confounder for perceived nasal appearance. However, no persistent neurosensory deficits were documented at the six-month follow-up when the survey was administered, reducing the likelihood that neurosensory alterations influenced breathing perception in this cohort.

Several factors may influence patient-reported perceptions, including individual expectations and prior experiences. In the present cohort, 32.5% of patients underwent bimaxillary surgery in addition to Le Fort I osteotomy. Mandibular repositioning may alter overall facial balance and potentially influence subjective nasal prominence and airway-related perceptions. Therefore, the inclusion of bimaxillary cases represents a potential confounding factor when interpreting patient-reported nasal aesthetic outcomes. This surgical heterogeneity should therefore be considered when interpreting the magnitude and direction of the reported perception changes. However, outcomes were not stratified according to surgical type, as the present study was designed to evaluate overall patient-reported perceptions rather than procedure-specific effects.

Furthermore, the direction and magnitude of maxillary repositioning (advancement and/or impaction) may differentially influence nasal base width, nasal tip projection, and upper airway dimensions. Although the present study did not stratify outcomes according to specific movement patterns, these biomechanical factors should be considered when interpreting patient-reported nasal aesthetic and breathing changes.

However, because structural modification of the nasal base primarily results from maxillary repositioning, Le Fort I osteotomy likely represents a major contributing factor to the observed perception changes within this treatment context.

The present study was designed as a cross-sectional survey administered at a standardized postoperative time point (six months), with the primary objective of evaluating patient-reported perceptions after surgical treatment. Therefore, retrospective assessment of preoperative perceptions was employed within this design framework rather than prospective baseline data collection.

However, perceptual adaptation may continue beyond the six-month postoperative period. Patient-reported satisfaction with facial aesthetics, particularly nasal appearance, can evolve over time as individuals further adjust to their altered facial identity and social feedback. While six months represents a clinically stable phase for soft tissue healing, longer-term psychological adaptation, expectation recalibration, or emerging dissatisfaction cannot be excluded. Therefore, the findings reflect perceptions at an early-to-intermediate postoperative stage rather than long-term outcomes. Longitudinal studies with extended follow-up would be valuable to determine whether the observed improvements remain stable, increase, or attenuate over time.

Another important limitation relates to the retrospective assessment of preoperative perceptions. Because preoperative evaluations were obtained postoperatively, participants’ recollection of their baseline perceptions may have been influenced by their surgical experience. Accordingly, the observed differences should not be interpreted as evidence of a direct causal effect of surgery but rather as associations between recalled preoperative and current postoperative perceptions. In particular, social perception outcomes may be especially vulnerable to recall and response bias, as postoperative satisfaction could have influenced how participants retrospectively interpreted feedback from their social environment prior to surgery.

Importantly, this issue extends beyond simple recall bias. Because preoperative perceptions were reconstructed after treatment, participants’ internal standards and evaluative frameworks may have changed over time. Such response-shift effects may occur when individuals recalibrate their interpretation of satisfaction, discomfort, or social impact following a major intervention. As a result, the way baseline status is remembered may be systematically altered. Consequently, the magnitude of perceived change may reflect not only differences between time points but also a change in the patients’ internal reference scale. This design feature should be considered when interpreting the extent of reported improvement.

All patients received routine preoperative orthodontic and surgical counseling as part of standard orthognathic treatment planning. Although no structured counseling protocol specifically targeting nasal breathing expectations was implemented for research purposes, preoperative information regarding potential functional changes may have influenced the postoperative perception of breathing outcomes. This factor should be considered when interpreting subjective differences between recalled preoperative and current postoperative breathing scores.

The questionnaire was researcher-developed and did not undergo formal psychometric validation or pilot testing prior to its application. Therefore, although the items were designed to reflect clinically relevant domains, the absence of prior validation procedures should be considered when interpreting the findings.

Furthermore, although demographic and clinical characteristics such as smoking status, alcohol consumption, antidepressant use, and the presence of systemic disease were reported descriptively to characterize the study population, no subgroup or multivariable analyses were performed to assess their potential influence on patient-reported outcomes. Therefore, possible confounding effects of these variables cannot be excluded.

The present study relied exclusively on patient-reported outcomes and did not incorporate objective assessments of nasal morphology or airway dimensions. This methodological choice reflects the primary aim of the investigation, which was to evaluate subjective perception rather than structural change. While objective measurements can provide valuable anatomical context, subjective aesthetic satisfaction and perceived breathing comfort do not always correspond directly to morphometric or volumetric findings. Accordingly, the results should be interpreted within a perception-based framework. Future studies integrating three-dimensional imaging or airway analysis with patient-reported outcomes may offer a more comprehensive understanding of the relationship between structural modification and perceived benefit.

Although orthognathic surgery can provide both aesthetic and functional benefits, concerns about complications, uncertainty, and cost may influence decision-making [36,37]. In the present study, 42.5% of participants retrospectively indicated that they would not have considered surgery solely for nasal appearance or breathing concerns. The observed postoperative shift in willingness responses, together with changes in reported aesthetic and functional perceptions, reflects the complex interplay between expectation, experience, and postoperative adaptation. These findings underscore that patient-reported outcomes in orthognathic surgery extend beyond structural modification and are shaped by individual interpretation and psychosocial context. Accordingly, comprehensive preoperative counseling and careful expectation management remain essential components of patient-centered care.

## 5. Conclusions

Le Fort I osteotomy was associated with more favorable patient-reported perceptions of nasal aesthetics and breathing when current postoperative evaluations were compared with retrospectively recalled preoperative perceptions at six months. These findings reflect differences in patient-reported perceptions within the framework of the present cross-sectional survey design.

Because preoperative perceptions were retrospectively reconstructed, findings should be interpreted as perception-based comparisons rather than objectively measured longitudinal changes.

Perceived outcomes may vary according to individual anatomical characteristics, surgical movements, and patient expectations. Therefore, comprehensive preoperative planning and detailed patient counseling that take individual differences and expectations into account remain important for optimizing both aesthetic and functional patient-centered outcomes.

## Figures and Tables

**Table 1:** Demographic and surgical characteristics of the study population.

**Variable**	**Group**	***n* (%)**
Gender	Male	85 (42.5)
	Female	115 (57.5)
Marital Status	Single	181 (90.5)
	Married	19 (9.5)
Systemic Disease	None	187 (93.5)
	Present	13 (6.5)
Regular Medication Use	None	190 (95.0)
	Present	10 (5.0)
Antidepressant Use	None	193 (96.5)
	Present	7 (3.5)
Smoking Status	None	130 (65.0)
	Present	70 (35.0)
Alcohol Consumption	None	176 (88.0)
	Present	24 (12.0)
Surgical Procedure	Isolated Le Fort I osteotomy Bimaxillary surgery (Le Fort I + mandibular osteotomy)	135 (67.5) 65 (32.5)
**Variable**	**Min–Max**	**Mean ± SD**
Age (years)	19–37	25 ± 4.19
Height (cm)	150–188	171.33 ± 8.87
Weight (kg)	42–130	72.96 ± 16.43

*n*: number of individuals; SD = standard deviation.

**Table 2:** Distribution of preoperative nasal aesthetic and breathing perception responses.

Questions	Definitely Not *n* (%)	Little *n* (%)	Neither a Little Nor a Lot *n* (%)	Quite a Lot *n* (%)	Definitely Yes *n* (%)
**Do you like the appearance of your nose ?**	17 (8.5)	73 (36.5)	47 (23.5)	42 (21.0)	21 (10.5)
**Do you think that you can breathe comfortably through your nose ?**	28 (14.0)	54 (27.0)	41 (20.5)	58 (29.0)	19 (9.5)
**Do you believe that your close environment and friends like the appearance of your nose ?**	40 (20.0)	33 (16.5)	77 (38.5)	38 (19.0)	12 (6.0)
**Do you think the current appearance of your nose negatively affects your social or professional life?** *	86 (43.0)	30 (15.0)	37 (18.5)	31 (15.5)	16 (8.0)
**Do you think your nose looks as good as it possibly can?**	26 (13.0)	70 (35.0)	77 (38.5)	21 (10.5)	6 (3.0)
**Before surgery did you consider undergoing surgery to change the appearance of your nose or to improve its breathing function?** ^†^	85 (42.5)	23 (11.5)	41 (20.5)	35 (17.5)	16 (8.0)

* Higher scores indicate a greater perceived negative impact. ^†^ Retrospective assessment of preoperative motivation, evaluated postoperatively.

**Table 3:** Distribution of postoperative nasal aesthetic and breathing perception responses.

Questions	Definitely Not *n* (%)	Little *n* (%)	Neither a Little Nor a Lot *n* (%)	Quite a Lot *n* (%)	Definitely Yes *n* (%)
**Do you like the appearance of your nose ?**	17 (8.5)	17 (8.5)	27 (13.5)	68 (34.0)	71 (35.5)
**Do you think that you can breathe comfortably through your nose ?**	19 (9.5)	18 (9.0)	34 (17.0)	67 (33.5)	62 (31.0)
**Do you believe that your close environment and friends like the appearance of your nose ?**	2 (1.0)	15 (7.5)	88 (44.0)	50 (25.0)	45 (22.5)
**Do you think the current appearance of your nose negatively affects your social or professional life?** *	59 (29.5)	22 (11.0)	52 (26.0)	64 (32.0)	3 (1.5)
**Do you think your nose looks as good as it possibly can?**	10 (5.0)	25 (12.5)	47 (23.5)	82 (41.0)	36 (18.0)
**After surgery do you still consider undergoing surgery to change the appearance of your nose or to improve its breathing function?** ^†^	21 (10.5)	13 (6.5)	39 (19.5)	97 (48.5)	30 (15.0)

* Higher scores indicate a greater perceived negative impact. ^†^ Postoperative perception reflecting residual motivation for further aesthetic or functional intervention.

**Table 4:** Comparison of preoperative and postoperative response scores.

Questions	Preoperative Med (Min–Max)	Postoperative Med (Min–Max)	*p*	r
Do you like the appearance of your nose?	2 (0–4)	3 (0–4)	<0.001 *	0.54
Do you think that you can breathe comfortably through your nose?	2 (0–4)	3 (0–4)	<0.001 *	0.47
Do you believe that your close environment and friends like the appearance of your nose?	2 (0–4)	3 (0–4)	<0.001 *	0.60
Do you think the current appearance of your nose negatively affects your social or professional life? ^†^	2 (0–4)	1 (0–4)	<0.001 *	0.24
Do you think your nose looks as good as it possibly can?	2 (0–4)	3 (0–4)	<0.001 *	0.66
Are you considering undergoing surgery to change the appearance of your nose or to improve its breathing function? ^‡^	1 (0–4)	3 (0–4)	<0.001 *	0.61

* Wilcoxon signed-rank test; statistical significance set at *p* < 0.05. r = Z/√n (non-zero differences); 0.10 = small, 0.30 = moderate, 0.50 = large effect size. ^†^ Higher scores indicate a greater perceived negative impact. ^‡^ Retrospective preoperative motivation compared with postoperative perception.

## Data Availability

The data presented in this study are available on request from the corresponding author. The data are not publicly available due to ethical and privacy restrictions, as the dataset includes patient-reported survey responses collected within the scope of an ethics committee-approved clinical study. Although the data are anonymized, public sharing is restricted to protect participant confidentiality in accordance with ethical guidelines.
